# A case report of anti‐NMDA receptor encephalitis in a 23‐year‐old female with acute psychiatric symptoms

**DOI:** 10.1002/ccr3.8870

**Published:** 2024-05-07

**Authors:** Elham Mohammed Khatrawi, Priyadarshi Prajjwal, Mohammed Dheyaa Marsool Marsool, Arpan Das, Abhijit Nagre, Pugazhendi Inban, Ali Dheyaa Marsool Marsool, Omniat Amir Hussin

**Affiliations:** ^1^ Department of Basic Medical Sciences College of Medicine, Taibah University Madinah Saudi Arabia; ^2^ Neurology Bharati Vidyapeeth Medical College Pune India; ^3^ University of Baghdad, Al‐Kindy College of Medicine Baghdad Iraq; ^4^ Internal Medicine RG Kar Medical College and Hospital Kolkata India; ^5^ Internal Medicine Topiwala National Medical College Mumbai India; ^6^ Internal Medicine Government Medical College Omandurar India; ^7^ Manhal University Khartoum Sudan

**Keywords:** anti‐NMDA receptor encephalitis, autoantibody, autoimmune encephalitis, cerebrospinal fluid, encephalitis

## Abstract

**Key Clinical Message:**

Prompt identification and management of anti‐N‐methyl‐D‐aspartate receptor encephalitis in young patients with acute psychiatric symptoms, seizures, and neurological deficits are crucial. Timely immunomodulatory therapy is essential for positive outcomes and minimizing long‐term complications. High suspicion for this rare disorder is necessary for timely diagnosis and optimal care.

**Abstract:**

Anti‐N‐methyl‐D‐aspartate (NMDA) receptor encephalitis is characterized by the presence of antibodies against the NMDA receptor, a crucial component of synaptic signaling. This autoimmune disorder often manifests with psychiatric symptoms, seizures, and neurological deficits. Early diagnosis is essential, as delayed treatment can result in severe complications. In this case, the patient received corticosteroids and intravenous immunoglobulin (IVIG), leading to a successful recovery with no lingering neurological abnormalities. The prompt initiation of treatment highlights the importance of recognizing this condition early. Anti‐NMDA receptor encephalitis is a rare autoimmune disorder that presents with a range of neurological symptoms. In this case report, we highlight the significance of early recognition and treatment by discussing the emergency room visit of a 23‐year‐old woman who presented with acute‐onset agitation, disorientation, and seizures. A 23‐year‐old woman, presented to the emergency room with acute‐onset agitation, disorientation, and seizures. Magnetic resonance imaging (MRI) scans revealed temporal lobe signal alterations and electroencephalogram (EEG) showed widespread activity slowing. Importantly, anti‐NMDA receptor antibodies were detected in both serum and cerebrospinal fluid, confirming the diagnosis of anti‐NMDA receptor encephalitis. This case report underscores the significance of understanding the presentation, diagnosis, and treatment of anti‐NMDA receptor encephalitis. Timely recognition and intervention are crucial for achieving favorable outcomes in patients with this rare but clinically important autoimmune disorder. Increased awareness among healthcare professionals is essential to ensure early diagnosis and prompt initiation of appropriate treatment strategies.

## 1 INTRODUCTION

Anti‐N‐methyl‐D‐aspartate (NMDA) receptor encephalitis is a rare autoimmune disease that affects the central nervous system.^1^ It is characterized by the production of antibodies against NMDA receptors, which are essential for synaptic plasticity as well as learning and memory processes in the brain. Although precise incidence rates are currently unknown, over 500 instances have been documented thus far. The California Encephalitis Project analyzed the cases of 761 people who were referred for encephalitis between September 2007 and February 2011. Among the cases with known causes, anti‐NMDA receptor encephalitis was the most common condition (32 out of 79 cases) in the group, occurring four times more frequently than herpes simplex‐type 1, West Nile virus, or varicella‐zoster virus.^1^ Patients with anti‐NMDA receptor encephalitis frequently have seizures, mobility issues, autonomic dysfunction, and psychiatric disorders.^2^ The long‐term outcomes of anti‐NMDA receptor encephalitis often result in substantial symptom relief by proper treatment, frequently improving baseline functional status or close to normal functioning. Nevertheless, specific individuals may encounter enduring cognitive impairments, motor abnormalities, or psychological manifestations, and a small percentage may undergo relapses that require continuous surveillance and treatment.^2^, ^3^ As this condition is commonly mistaken for a mental disorder or viral encephalitis, early detection, and treatment are crucial. The aim of this case report is to raise awareness of this rare but important autoimmune disorder and its clinical manifestations. By presenting this case of a patient with anti‐NMDA receptor encephalitis, the report aims to provide clinicians with a better understanding of this condition's presentation, diagnosis, and treatment.

## 2 CASE PRESENTATION

A 23‐year‐old female patient came to the emergency department with complaints of convulsions, agitation, and rapid onset disorientation. The patient had an unremarkable medical history and no history of seizures, and substance or alcohol addiction. The initial assessment of vital signs indicated a stable hemodynamic status with normal measurements of blood pressure, heart rate, and respiration rate. Nevertheless, additional evaluation using the Glasgow Coma Scale revealed a diminished state of awareness, with a score of 12 out of 15. Upon further investigations, the patient mentioned that 3 weeks ago, she was diagnosed with a mild viral illness, with a dry cough and rhinitis as the main symptoms. The patient was noticed to have fluctuating degrees of cognition, confusion, and disorientation along with uncontrollable limb and face muscle movements.

Blood testing comprising a full metabolic panel, thyroid function tests, viral serology, and complete blood count showed no obvious abnormalities. Following the initial presentation, the patient's condition deteriorated, with escalating agitation and psychosis‐like symptoms, including auditory hallucinations, paranoid delusions, and disorganized thought processes. She exhibited erratic behavior and displayed signs of emotional dysregulation, which, of notice, were her extreme mood swings. After consultation with the psychiatry department, antipsychotic medication in the form of risperidone was initiated at a dose of 1 mg orally twice daily. The patient showed no significant improvement in neurological symptoms, so a lumbar puncture was obtained. This came back positive for anti‐NMDA receptor antibodies and oligoclonal bands. Other than the positive results from the serum and CSF anti‐NMDA receptor antibody test (Table 1) and the study of the cerebral fluid, which revealed lymphocytic pleocytosis with a preponderance of CD4+ T cells, the laboratory results were unremarkable (Table 2).

**TABLE 1:** The laboratory findings were unremarkable, except for the positive serum anti‐N‐methyl‐D‐aspartate (NMDA) receptor antibody testing, which was suggestive of anti‐NMDA receptor encephalitis.

Test	Results	Reference range
Complete blood count		
Hemoglobin	13.8 g/dL	12.0–15.5 g/dL
Hematocrit	41.2%	36.0%–46.0%
White blood cell count	7.5 × 10^9/L	4.5–11.0 × 10^9/L
Platelet count	200 × 10^/L	150–450 × 10^9/L
Comprehensive metabolic panel		
Sodium	138 mm/L	135–145 mm/L
Potassium	4.2 mmol/L	3.5–5.0 mmol/L
Chloride	100 mmol/L	98–106 mmol/L
Bicarbonate	24 mmol/L	22–32 mmol/L
Blood urea nitrogen	12 mg/dL	7–25 mg/dL
Creatinine	0.9 mg/dL	0.5–1.2 mg/dL
Glucose	94 mg/dL	70–100 mg/dL
Thyroid function tests		
Thyroid stimulation hormone (TSH)	2.5 μIU/mL	0.4–4.5 μIU/mL
Free thyroxine (FT4)	1.2 ng/dL	0.8–1.8 ng/dL
Viral serology		
Herpes simplex virus 1 & 2 IgG	Negative	
Varicella‐zoster virus IgG	Positive	
Serum Anti‐NMDA receptor antibody	Positive	

**TABLE 2:** The CSF analysis showed a lymphocytic pleocytosis with a predominance of CD4+ T cells, elevated protein levels, and normal glucose levels. This finding is consistent with autoimmune encephalitis, particularly anti‐N‐methyl‐D‐aspartate (NMDA) receptor encephalitis.

Test	Result
Cell count	cells/μL
Red blood cells	0 cells/μL
White blood cells	30 cells/μL
Differential cell count	
Lymphocytes	85%
Monocytes	10%
Neutrophils	5%
Protein	100 mg/dL
Glucose	65 mg/dL
Anti‐NMDA receptor antibodies	Positive

### 2.1 Differential diagnosis

**FIGURE 1:**
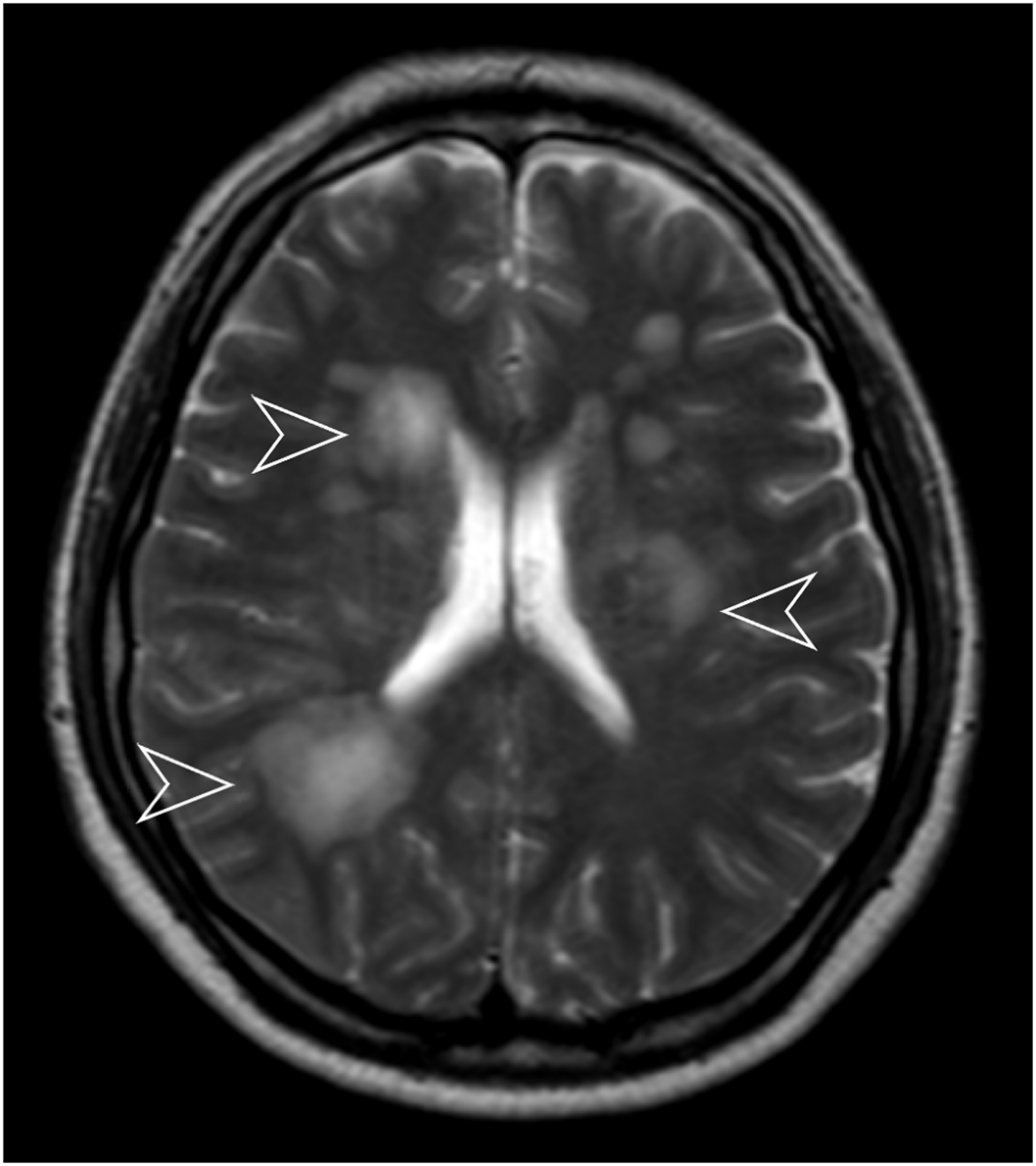
Axial T2 magnetic resonance imaging showing high T2 signal changes that are multifocal and bilateral multifocal bilateral high T2 signal changes in the periventricular deep white matter (arrowheads).

**FIGURE 2:**
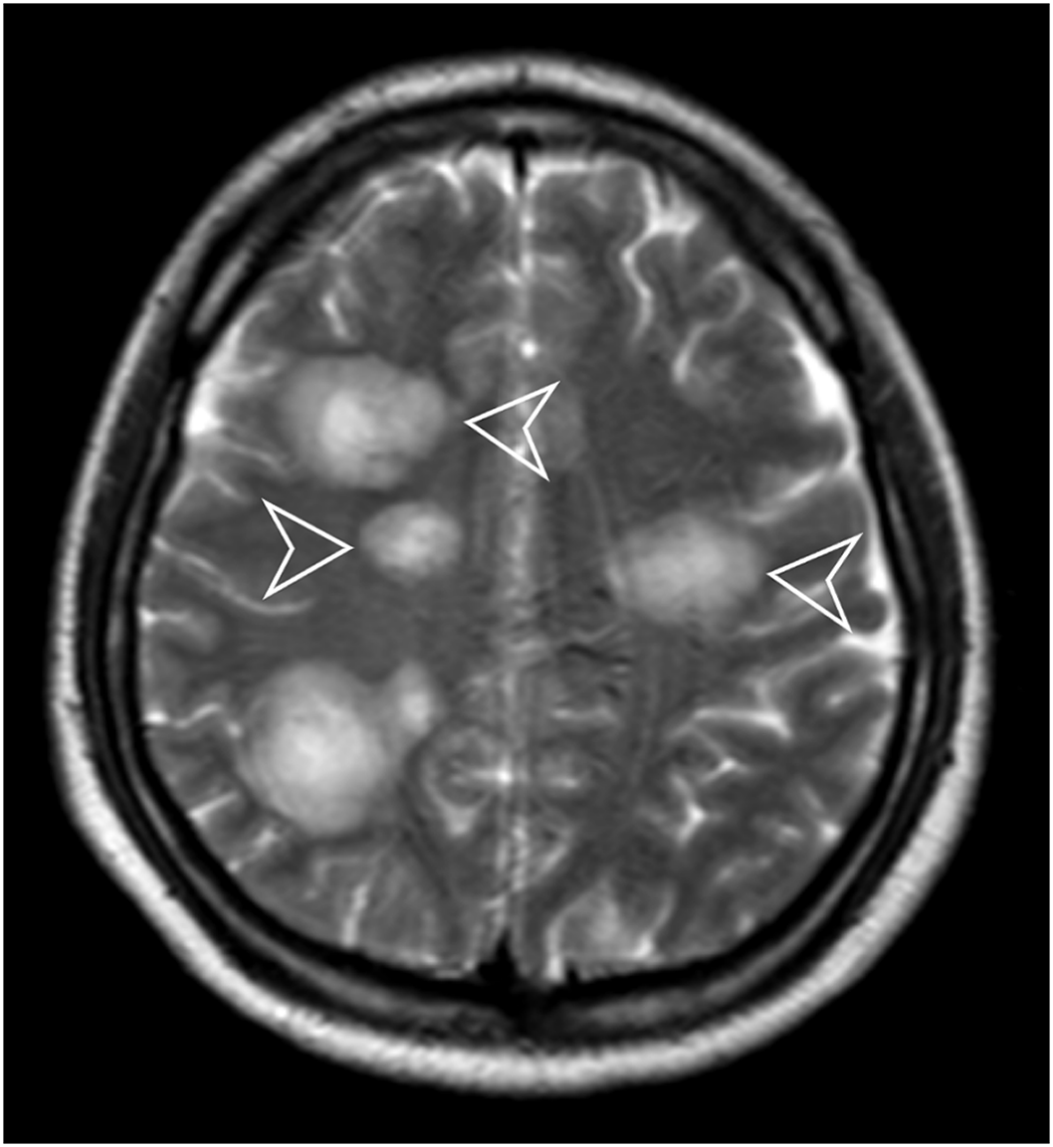
Axial T2 magnetic resonance imaging showing high T2 signal changes that are multifocal and bilateral in the bilateral basal nuclei (arrowheads) with surrounding vasogenic edema.

Magnetic resonance imaging (MRI) of the brain showed diffuse high signal T2 surrounding the ventricles (Figure 1) and the basal nuclei (Figure 2) bilaterally with surrounding vasogenic edema. The final diagnosis of anti‐NMDA receptor encephalitis was made after the exclusion of other psychiatric and immune‐mediated disorders. The rationale for reaching the diagnosis was the high suspicion index seen in the patient. This includes the viral‐like prodrome seen in the patient, the presence of rapid/acute onset of symptoms such as seizures, decreased level of consciousness, slurred speech, and abnormal posture and movements, in the previously normal female along with the presence of pleocytosis, oligoclonal bands, and anti‐NMDA receptor positivity.

### 2.2 Outcomes and follow‐up

The patient was started on intravenous immunoglobulin (IVIG) therapy at 2 g/kg over 5 days before beginning maintenance treatment with the monoclonal antibody rituximab, and high‐dose corticosteroids (methylprednisolone). She was also given antiepileptic medication to control her seizures. The patient's neurological symptoms gradually improved over the next few days. She was transferred to the neurology department for further management.

**FIGURE 3:**
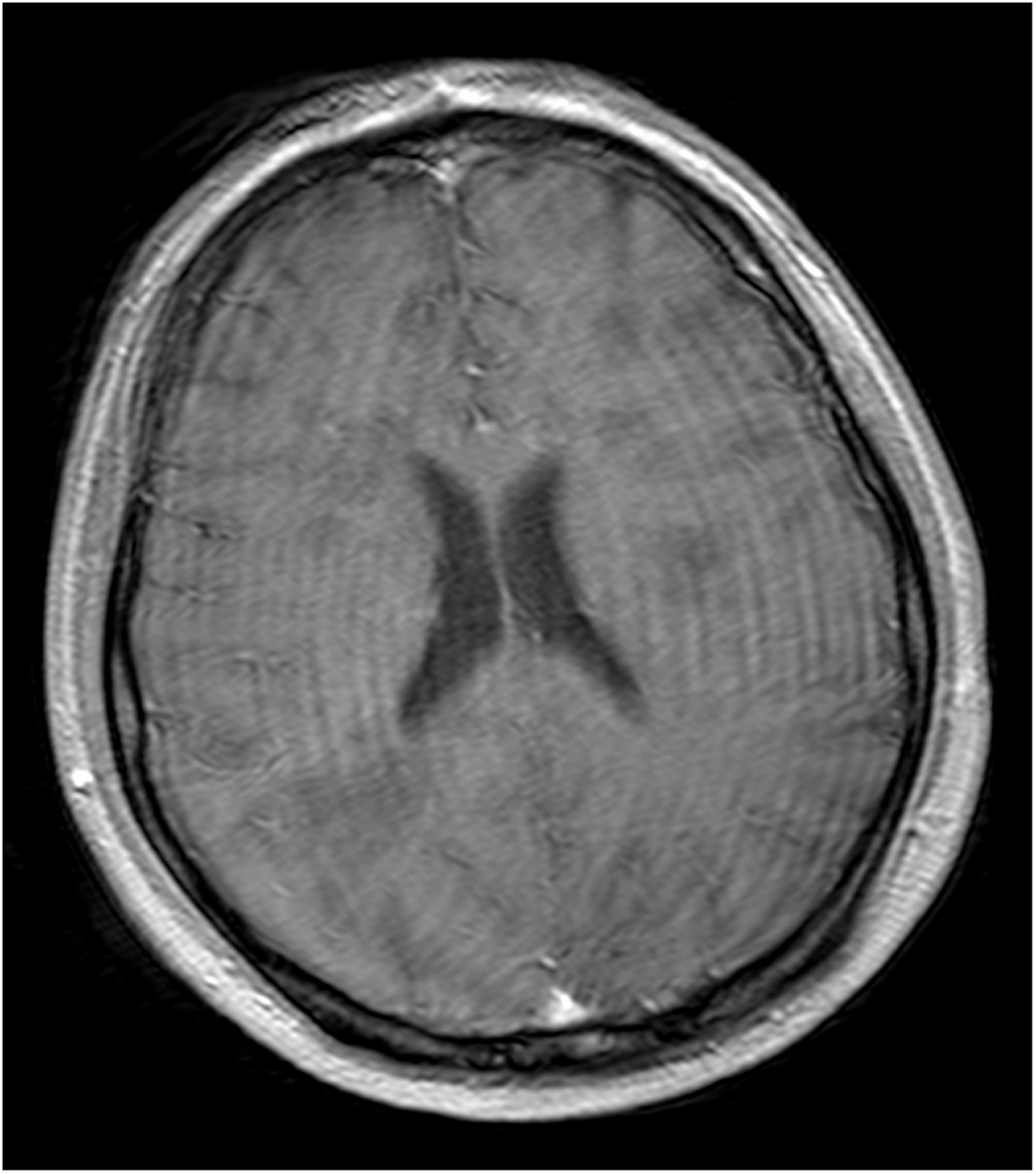
Magnetic resonance imaging with contrast done 5 days post‐treatment with intravenous immunoglobulin, IV methylprednisolone, and rituximab. Showing that the post‐contrast T1 enhancement in the nodular lesions resolved.

The patient showed remarkable clinical improvement in the following few weeks. A subsequent MRI revealed that the previously noted anomalies had disappeared (Figure 3). After being released from the hospital, the patient received routine follow‐up care at the outpatient clinic. Also, after a follow‐up of 23 days after onset, CSF analysis showed the disappearance of anti‐NMDA receptor antibodies.

## 3 DISCUSSION

NMDA receptor antibodies positivity is a definitive sign of the autoimmune disease anti‐NMDA receptor encephalitis in the brain.^1^ With a median age of onset of 21 years, the disease primarily affects young women, and the prodrome frequently resembles a viral illness.^2^ Anti‐NMDA receptor encephalitis often manifests as a combination of neurological symptoms, including seizures, movement problems, and autonomic dysfunction, as well as psychological symptoms like agitation, anxiety, and hallucinations. A combination of these symptoms may occasionally be present in individuals, raising the possibility that they are suffering from another disorder such as viral encephalitis, schizophrenia, or neuroleptic malignant syndrome.^2^, ^3^

On physical examination, patients with anti‐NMDA receptor encephalitis may have abnormal movements such as choreoathetosis or dystonia, as well as autonomic instability and decreased level of consciousness. However, physical findings may be normal in some cases.^3^

A lymphocytic pleocytosis in the CSF and higher levels of inflammatory markers like CRP and ESR are common signs of inflammation that can be found. Imaging tests such as electroencephalograms (EEG) and MRI of the brain may also reveal anomalies, such as signal alterations in the temporal lobes or a generalized slowing of EEG activity.^2^, ^3^, ^4^

Clinical symptoms, CSF pleocytosis, and the presence of serum or CSF antibodies against the NMDA receptor are used to make the diagnosis of anti‐NMDA receptor encephalitis. In order to meet the diagnostic standards outlined by Graus et al. in 2016,^4^, ^6^ at least four out of six key criteria must be present, including mental symptoms, neurological symptoms, seizures, movement problems, autonomic dysfunction, and CSF pleocytosis.

In anti‐NMDA receptor encephalitis, the characteristic radiological manifestation on MRI often includes multifocal and bilateral high T2 signal changes surrounding the ventricles and basal nuclei, accompanied by surrounding vasogenic edema.^7^ Comparatively, other MRI patterns seen in autoimmune encephalitis may vary depending on the specific underlying autoimmune process.^7^, ^8^ For example, in limbic encephalitis, there may be hyperintense signal abnormalities in the medial temporal lobes on fluid‐attenuated inversion recovery sequences, often involving the hippocampi bilaterally. In contrast, autoimmune encephalitis associated with antibodies targeting neuronal cell‐surface antigens, such as LGI1 or CASPR2, may show more localized or asymmetric MRI findings, often involving specific brain regions such as the medial temporal lobes or limbic structures.^8^

In addition to immunosuppression with corticosteroids, IVIG, or plasmapheresis, anti‐NMDA receptor encephalitis is treated by removing any underlying tumors that may be present.^5^, ^6^ Immunotherapy with cyclophosphamide or rituximab may be required in extreme situations. Early treatment is essential since treatment delays have been linked to worse outcomes and increased rates of recurrence.^9^, ^10^

## 4 CONCLUSION

Our case highlights the significance of considering anti‐NMDA receptor encephalitis in young patients who experience sudden psychiatric symptoms and changes in mental state, even if they have no previous history of neurological issues. Timely diagnosis and prompt starting of immunotherapy are essential for achieving positive results. Further investigation should prioritize comprehending the processes of the disease, identifying early markers, and creating standardized treatment procedures to enhance patient care and outcomes. Healthcare personnel must have heightened awareness in order to promptly recognize and intervene in this potentially incapacitating disease.

## AUTHOR CONTRIBUTIONS

**Elham Mohammed Khatrawi:** Methodology; writing – original draft; writing – review and editing. **Priyadarshi Prajjwal:** Methodology; writing – original draft; writing – review and editing. **Mohammed Dheyaa Marsool Marsool:** Conceptualization; resources; validation; writing – original draft; writing – review and editing. **Arpan Das:** Visualization; writing – original draft. **Abhijit Nagre:** Writing – original draft; writing – review and editing. **Pugazhendi Inban:** Writing – original draft; writing – review and editing. **Ali Dheyaa Marsool Marsool:** Writing – original draft; writing – review and editing. **Omniat Amir Hussin:** Investigation; writing – original draft.

## FUNDING INFORMATION

None.

## CONFLICT OF INTEREST STATEMENT

The authors declare no conflicts of interest, financial or otherwise.

## ETHICS STATEMENT

Not applicable.

## CONSENT

The authors certify that they have obtained written informed consent from the patient to publish this report in accordance with the journal's patient consent policy. This report does not contain any personal information that could lead to the identification of the patient.

## Data Availability

The data that support the findings of this study are available within the manuscript itself.
